# The effect of increasing temperature on simulated nanocomposites reinforced with SWBNNs and its effect on characteristics related to mechanics and the physical attributes using the MDs approach

**DOI:** 10.1016/j.heliyon.2023.e21022

**Published:** 2023-10-14

**Authors:** Somayeh Tavasolikejani, Ashkan Farazin

**Affiliations:** a Isfahan University of Technology, Department of Chemistry, Isfahan, 8415683111, Iran; b Department of Solid Mechanics, Faculty of Mechanical Engineering, University of Kashan, P.O. Box 87317-53153, Kashan, Iran

**Keywords:** Molecular dynamics simulation, Radial distribution function, Nanocomposite, Single-walled boron nitride nanotubes, Mechanical properties

## Abstract

This study examines the effect of increasing temperature (300, 350, 400, 450 and 500 K) on simulated nanocomposites reinforced with exploration of the impact of single-walled boron nitride nanotubes (SWBNNTs) on both the mechanical properties (including Young's modulus, Poisson's ratio, shear modulus, and bulk modulus) and the physical property of density, achieved through molecular dynamics (MDs) simulations. MDs utilized to simulate nanocomposite models consisting of five case studies of SWBNNs with different chiralities (5, 0), (10, 0), (15, 0), (20, 0), and (25, 0) as the reinforcement and using thermoplastic polyurethane (TPU) as the common matrix. The results reveal that with increasing temperature and chiralities of SWBNNTs, the density and Poisson's ratio increase dramatically, and Young's, shear, and bulk moduli decrease continuously. At a consistent temperature, there is a noteworthy trend in the mechanical properties of SWBNNTs with various chiralities. This includes the increase in Young's modulus, Poisson's ratio, shear modulus, and bulk modulus in the simulated nanocomposite, ranging from SWBNNTs (5, 0) to (25, 0). Similarly, the physical property of density exhibits an increasing trend from SWBNNTs (5, 0) to (20, 0) and then decreases at SWBNNTs (25, 0). To validate the accuracy of these findings, a Radial Distribution Function (RDF) diagram is generated using Materials Studio software.

## 1 Introduction

Boron nitride nanotubes (BNNTs) are slender, low-mass, and robust nanostructures, exhibiting exceptional stability attributed to their honeycomb-shaped lattice formed by sp^2^ bonding [1,2], these nanostructures have excellent physical, mechanical, and thermal properties. So in recent years, many researchers have turned their attention to it [[3], [4], [5]]. BNNTs are considered to be tubes wrapped in a hexagonal nitride plate. BNNTs are better biocompatible than carbon nanotubes (CNTs) [[6], [7], [8]]. The extensive application of these nanomaterials in disease diagnostics and treatment techniques has been facilitated by their remarkable biocompatibility and non-hazardous characteristics [[9], [10], [11], [12]]. Despite the good properties of CNTs due to their toxicity, they are almost non-existent for drug delivery in biological systems [[13], [14], [15], [16], [17], [18]]. Therefore, BNNTs were considered as a suitable alternative [19]. Due to their non-toxicity, high biocompatibility, and unique properties, such as magnetic properties by various molecules, BNNTs are highly applicable in medical sciences for therapeutic purposes and diagnosis [[20], [21], [22], [23], [24]]. In addition to their resistance to decomposition in vacuum, air, and some liquids, BNNT nanostructures have significant resistance to high-temperature oxidation compared to CNTs. The oxidation temperature of BNNTs is 800 °C [25], while for CNTs are equal to 400 °C [26]. This high level of resistance of BNNTs to oxidation allows them to be used at high temperatures [27]. The impressive thermal conductivity and notable electrical insulating characteristics of BNNTs position them as a prospective option for future applications in thermal interface materials [28]. In the last few years, much attention has been paid to the thermal interface materials based on CNTs. Also, CNTs can be metallic or semiconductor-based on chirality. Metal nanotubes are conductive and can interfere electrically with microelectronic components, but semiconductor nanotubes are electrical insulators and are very suitable for thermal interface materials and use in nanofluids [29]. BNNTs are also divided into three important categories according to the arrangement of nitrogen and boron atoms in the pipe section: Armchair, Chiral, and Zigzag [[30], [31], [32]]. The MDs method is faster and more accurate than computational methods and can be used for complex systems with high atomic numbers [33]. Hence, in this study, we have employed the MDs technique to forecast the physical and mechanical attributes at various temperatures. Several researchers have also employed MDs for modeling nanocomposite samples at the nanoscale [[34], [35], [36], [37], [38]]. Choyal et al. [39], They discovered that the electronic characteristics of BNNTs can be modified through various means, including changes in loading conditions, diameter, and vacancy concentration. This fundamental research underscores the crucial influence of vacancy-defected BNNTs on shaping their mechanical and electronic properties, as they find extensive utility in a wide range of applications, including nano-electronic devices and strengthening components in versatile nanocomposites. Choyal et al. [40], observed that the mechanical characteristics and failure response of MWBNNTs are markedly influenced by factors such as the number of layers, chirality, and temperature. The outcomes of their research can be applied in designing advanced nanocomposite structures using MWBNNTs tailored for specific applications in thermal conditions. In Choyal et al.'s study [41], they noted that zigzag BNNTs exhibit stronger mechanical behavior when compared to armchair configurations. Furthermore, it was determined that the mechanical properties of BNNTs are contingent upon chirality and diameter, with a pronounced impact on smaller diameter tubes.

Shahshahani et al. [42], In their study, the researchers employed molecular dynamics (MD) simulations to explore the thermal (TP) and mechanical properties (MP) of methacrylic acid porous hydrogels within an aqueous environment. The findings revealed that as the volume fraction (VF) increased from 5 % to 15 %, the thermal conductivity (TC) of the hydrogel also increased, going from 0.103 to 0.114 W/m·K. Furthermore, the Young's modulus (YM) and ultimate strength (US) demonstrated an increase, rising from 247 MPa to 131–274 MPa and 146 MPa, respectively.

In a separate work, Koochaki et al. [43] conducted molecular dynamics (MD) simulations to analyze hydrogel-cellulose nanocomposites under standard conditions. The simulation outcomes indicated the physical stability of the samples at 300 K and 1 bar.

Mahjoori et al. [44], Their research focused on investigating the influence of different percentages and sizes of Mg nanoparticles (NPs) on the thermal and mechanical properties of reinforced cement using molecular dynamics (MD) simulations. To assess these effects, changes in Young's modulus (YM), maximum temperature (MT), and ultimate strength (US) were analyzed. As a result, the reinforced cement sample exhibited enhancements in US, YM, and MT, with values increasing from 0.879 to 1.171 MPa, 1.326 MPa, and 0.255 MPa, respectively, when the NPs percentage was raised to 4 %, elevating the temperature from 1321 K to 1403 K.

Fada et al. [45], The study focused on assessing the mechanical characteristics of a multi-component scaffold with varying levels of porosity. Mori-Tanaka equations were employed to estimate different attributes of calcium phosphate (CaP) bone cement reinforced with strontium nitrate nanoparticles (NPs), taking into account porosity percentages before and after exposure to a simulated body fluid (SBF) solution. The analysis of mechanical properties revealed an increase in grain and particle size following the addition of nitrate to the bone cement.

Liang et al. [46], In their research, they assessed the elastic modulus and hardness of fused silica and then replicated the findings through molecular dynamics (MD) and finite element analysis (FEA). In their investigation, MD was employed to model the mechanical behavior of fused silica structures with atomic-level precision, with initial conditions set at T0 = 300 K and P0 = 1 bar. Using this MD simulation, they were able to determine various mechanical constants, including hardness (measured with a Berkovich tip), elastic modulus (measured with a Berkovich tip), and fracture toughness (measured with a Cube Corner tip) for the fused silica nanostructure. The simulation indicated that the hardness of the atomic matrix was measured at 8.84 GPa.

Al-Haik et al. [47] investigated how the structural aspects of carbon nanotubes (CNTs) affect the radiation-induced damage in composites composed of PE and SWCNTs with varying chiralities using molecular dynamics (MD). In another study by Farazin et al. [48], they analyzed the mechanical performance of a porous bio-nanocomposite through MD simulations to compare the outcomes with experimental data. Their findings suggested that certain theories, such as Dewey's method, do not accurately predict the mechanical properties of porous nanocomposites. Alian et al. [49], They explored the interfacial and mechanical characteristics of an epoxy composite reinforced with carbon nanotubes (CNTs). The study proceeded in two phases. In the initial phase, they employed molecular dynamics simulations to analyze the atomic-level interfacial and mechanical properties of a representative volume element (RVE) with transversely isotropic characteristics, made up of a composite of CNTs and epoxy.

Reviewing existing literature, it becomes evident that prior studies have predominantly concentrated on the mechanical properties of epoxy resin reinforced with carbon nanotubes (CNTs). This article represents a novel approach by investigating the influence of elevated temperatures (ranging from 300 K to 500 K) on simulated nanocomposites strengthened with single-walled boron nitride nanotubes (SWBNNTs). The focus is on evaluating the resulting impact on both mechanical properties (Young's modulus, Poisson's ratio, shear modulus, and bulk modulus) and physical properties (density) using the molecular dynamics (MD) methodology. The MD simulations are employed to model nanocomposites, featuring five distinct case studies of SWBNNTs with different chiralities (5, 0), (10, 0), (15, 0), (20, 0), and (25, 0), as the reinforcement. These nanocomposites utilize thermoplastic polyurethane (TPU) as the matrix material, known for its compatibility with biological applications.

## 2 Materials and methods

### 2.1 MDs simulations

**Fig. 1:**
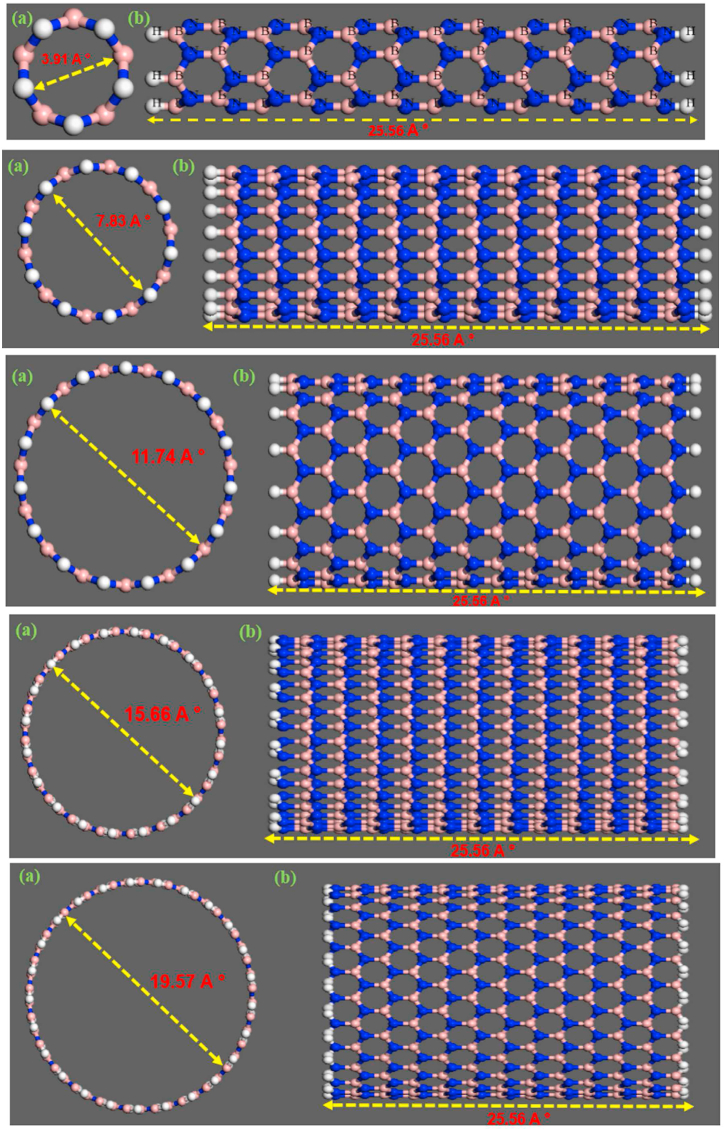
The view of (a) Diameter (b) Length of armchair SWBNNTs with 6 repeat units in Materials Studio software.

In this study, we utilized Materials Studio software version 8.0 for conducting molecular dynamics (MD) simulations. Our objective was to explore and predict the mechanical properties (Young's modulus, Poisson's ratio, shear modulus, and bulk modulus) as well as physical properties (density) of armchair single-walled boron nitride nanotubes (SWBNNTs) with various chiralities, including (5, 0), (10, 0), (15, 0), (20, 0), and (25, 0). These SWBNNTs had different diameters, specifically 3.91, 7.83, 11.74, 15.66, and 19.57 Å, but were of the same length, measuring 25.56 Å, as depicted in Fig. 1. To prevent the influence of unsaturated boundary conditions, hydrogen atoms terminated both ends of the SWBNNTs. It is essential to note that the bond length between the atoms was set at 1.42 Å.

For the prediction of mechanical and physical properties, we employed the Universal force field, which is a commonly used force field for defining atomic interactions, both intermolecular and intramolecular, within the MD framework. The simulation was conducted in three stages, namely NVE (microcanonical ensemble), NVT (canonical ensemble), and NPT (isothermal-isobaric ensemble), to determine the final properties of these simulated nanocomposites, as outlined below:

In the microcanonical (NVE) ensemble, the system remains isolated from changes in moles (N), volume (V), and energy (E). This corresponds to an adiabatic process where there is no heat exchange. A molecular dynamics trajectory in the NVE ensemble is characterized by an exchange of potential and kinetic energy, with the total energy being conserved.

In the canonical (NVT) ensemble, the number of particles (N), volume (V), and temperature (T) are conserved. It is sometimes referred to as constant temperature molecular dynamics (CTMD). In NVT, energy is exchanged with a thermostat during endothermic and exothermic processes. The purpose of NVT is to provide the system with the energy required to reach equilibrium, releasing the initial internal stresses applied during the simulation box construction. During this step, the simulation box was equilibrated at temperatures of 300, 350, 400, 450, and 500 K under the NVE conditions. A simulation time of 100 ps was found to be sufficient to reach equilibrium in the MD simulations.

The NPT ensemble helps eliminate any remaining stresses within the system. In this phase, the system was subjected to a pressure of 1 atm and temperatures of 300, 350, 400, 450, and 500 K under NPT conditions. The simulation duration at this stage was set to 100 ps.

**Fig. 2:**
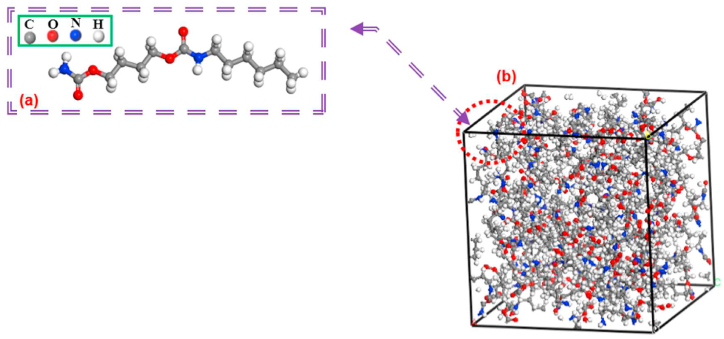
(a) A monomer of TPU (b) Simulated Polymer of TPU with 100 chain length.

This article employs a single chain of thermoplastic polymer consisting of 100 polymer units of polyurethane (TPU) as the matrix, as illustrated in Fig. 2 [50]. TPU is a material of significant importance for medical implants due to its excellent mechanical properties, including high tensile strength, toughness, resistance to abrasion, and durability, as well as its strong biocompatibility [51,52]. These attributes categorize TPUs as highly suitable materials for medical applications. Polyurethane elastomers exhibit superior mechanical compatibility, high tensile strength, and minimal loss of elasticity. Laboratory investigations have demonstrated that urethane elastomers possess exceptional chemical and mechanical characteristics, making them preferable for vascular prostheses when compared to other polymers commonly used in cardiovascular implants. In animal testing research, biodegradable polyurethane foam reinforced with SWBNNTs has shown good biocompatibility [53].

**Fig. 3:**
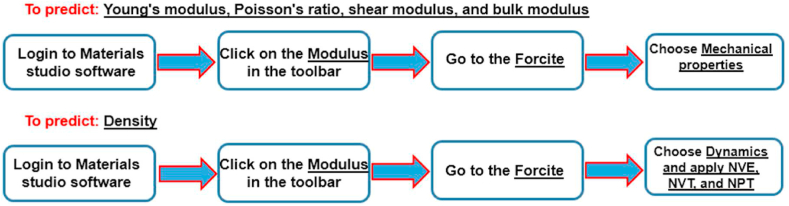
Four stages for obtaining significant mechanical and physical characteristics using the MDs technique.

The following steps are done to complete the MDs method to achieve significant mechanical and physical properties as presented in Fig. 3.

### 2.2 Simulation methodology

**Fig. 4:**
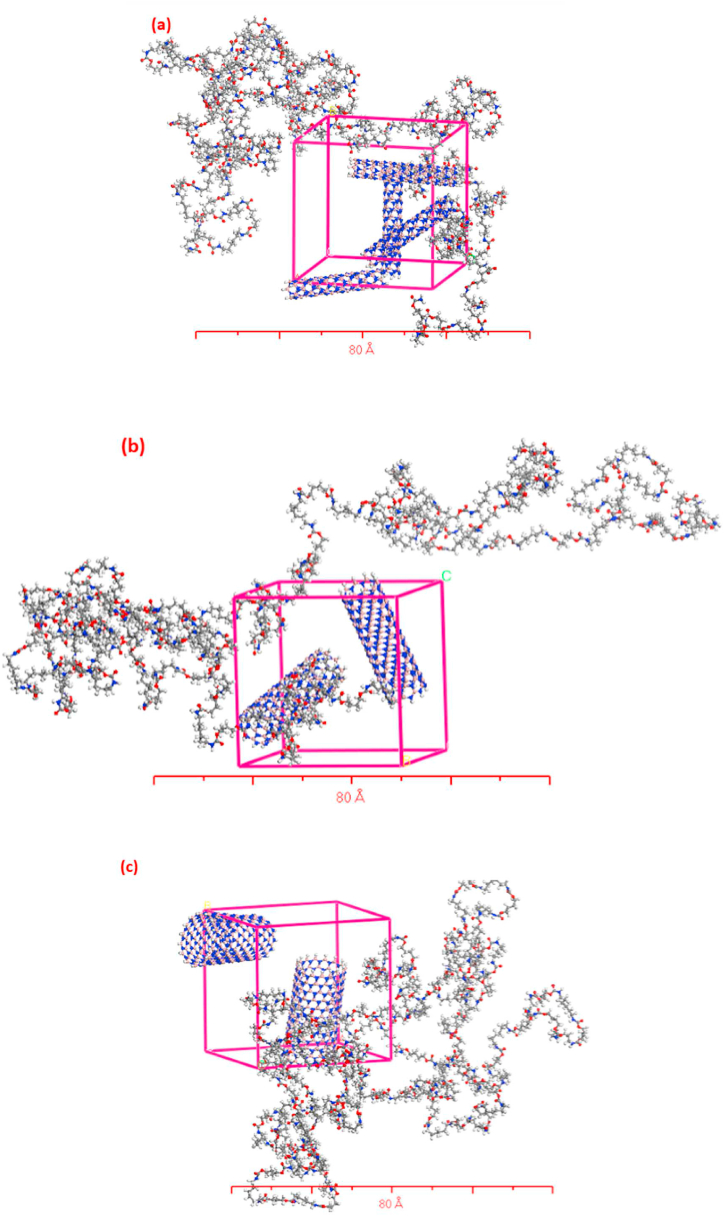

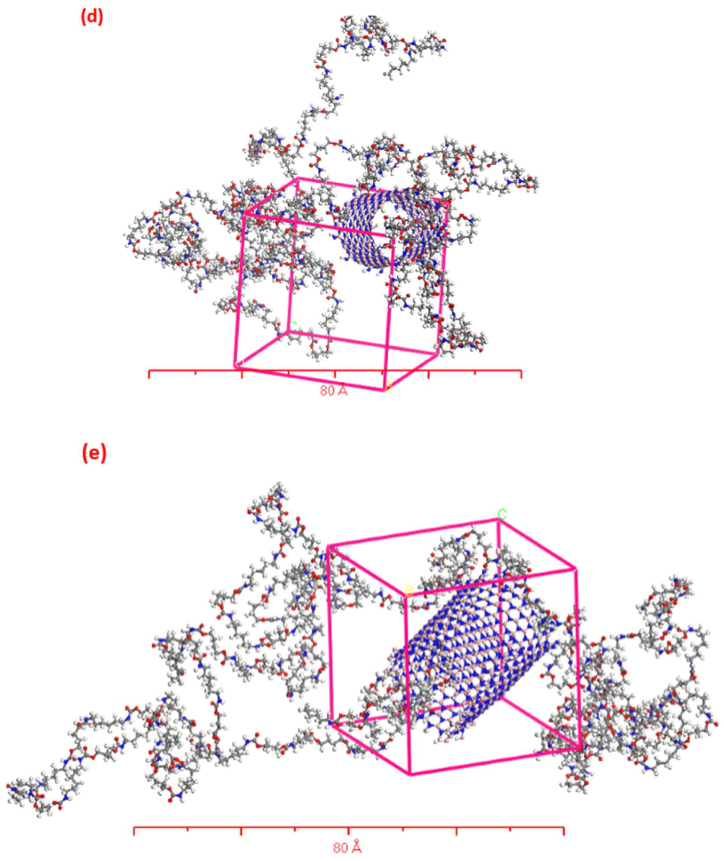
Five simulated nanocomposite boxes with different chiralities (a) (5, 0), (b) (10, 0), (c) (15, 0), (d) (20, 0), and (e) (25, 0) of SWBNNTs-TPU using Materials Studio software.

In this section, we investigate the impact of elevated temperatures (300, 350, 400, 450, and 500 K) on simulated nanocomposite structures reinforced with single-walled boron nitride nanotubes (SWBNNTs), and its consequent effect on their mechanical properties (Young's modulus, Poisson's ratio, shear modulus, and bulk modulus), as well as their physical properties (density), as illustrated in Fig. 4. To perform these simulations in the MDs software with precision, we maintained a consistent reinforcement weight of 25 % of the total weight, while the polymer content remained constant at 75 %. It's important to note that the overall weight of the simulated composites was set at 10 g, with the polymer (TPU) accounting for 7.5 g. Table 1 provides comprehensive molecular details for each of the five case studies, including the SWBNNT type, inner radius, number of atoms in each SWBNNT, total atom count, and lattice dimensions.

**Table 1:** Details of five case study for simulated nanocomposites boxes (SWBNNTs-TPU).

Rolling Procedure	**Type of SWBNNTs**	**Inner radius (Å)**	**Number of each SWBNNT atoms**	**No. of total atoms**	Lattice **Dimensions (Å)**
**Armchair**	(5,0)	3.91	130	3322	34.2 × 34.2 × 34.2
**Armchair**	(10,0)	7.83	260	3464	34.8 × 34.8 × 34.8
**Armchair**	(15,0)	11.74	390	3582	35.6 × 35.6 × 35.6
**Armchair**	(20,0)	15.66	520	3652	36.2 × 36.2 × 36.2
**Armchair**	(25,0)	19.57	650	3758	37.9 × 37.9 × 37.9

## 3 Result and discussion

### 3.1 Results of physical properties (density) by MD

**Fig. 5:**
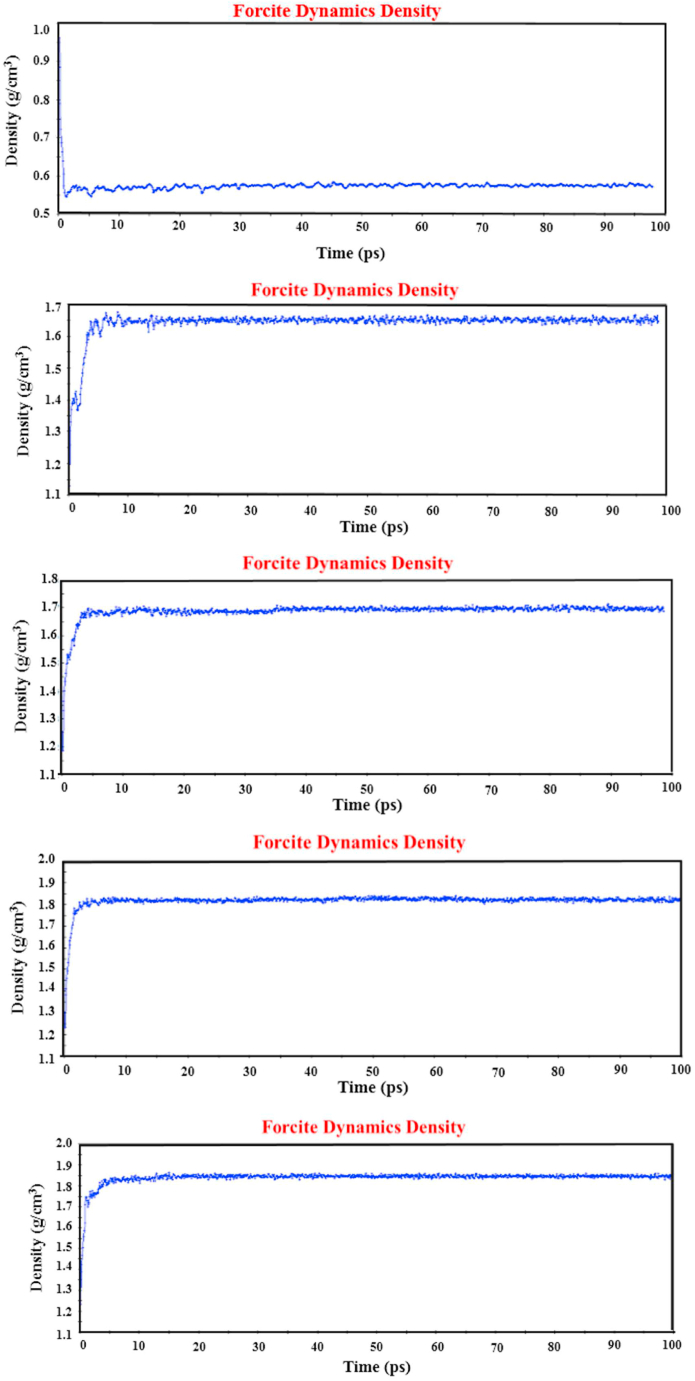
Density diagram armchair SWBNNTs (25, 0) mixed with TPU.

Density is a crucial physical property when it comes to atomic modeling. It serves as an indicator of how accurately atoms are positioned at their equilibrium distances. When atomic modeling is carried out correctly, the density of the atomic system should closely match the actual density of the system at the macro scale. Additionally, since various ensembles are employed in simulations, leading to changes in atomic conditions, it is expected that the density value will converge over the simulation time. Therefore, the convergence of system properties, including density, serves as an indicator of the accuracy of the atomic modeling process. In this article, we investigate the impact of increasing temperature on significant mechanical and physical properties. To streamline the presentation, we have selected SWBNNTs with chirality (25, 0) for this section, and we have created density diagrams at different temperatures (300, 350, 400, 450, and 500 K). To construct the density diagram as described above, we first employ the NVT ensemble to equilibrate the system's energy, followed by the NPT ensemble to generate the density diagram. As shown in Fig. 5, the predicted densities are approximately 0.55, 1.65, 1.69, 1.80, and 1.85 g/cm³ for temperatures of 300, 350, 400, 450, and 500 K, respectively.

### 3.2 Diagram of Radial Distribution Function

**Fig. 6:**
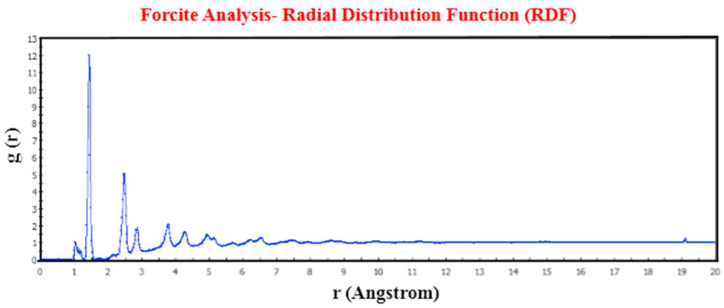
RDF of SWBNNTs with chirality (25, 0) mixed with TPU convergence to the number 1.

The Radial Distribution Function (RDF) stands as a critical metric in assessing the equilibrium accuracy of molecular dynamics simulations. In the case of solids or composites, the RDF should ultimately converge to a value of "1," serving as a precise indicator for achieving atomic equilibrium in solid materials. To streamline the presentation, we have generated the RDF for SWBNNTs with chirality (25, 0) using TPU. Fig. 6 illustrates that the RDF diagram consistently converges to the value of "1," demonstrating the high level of accuracy achieved in the simulation.

### 3.3 Simulation of mechanical properties

**Fig. 7:**
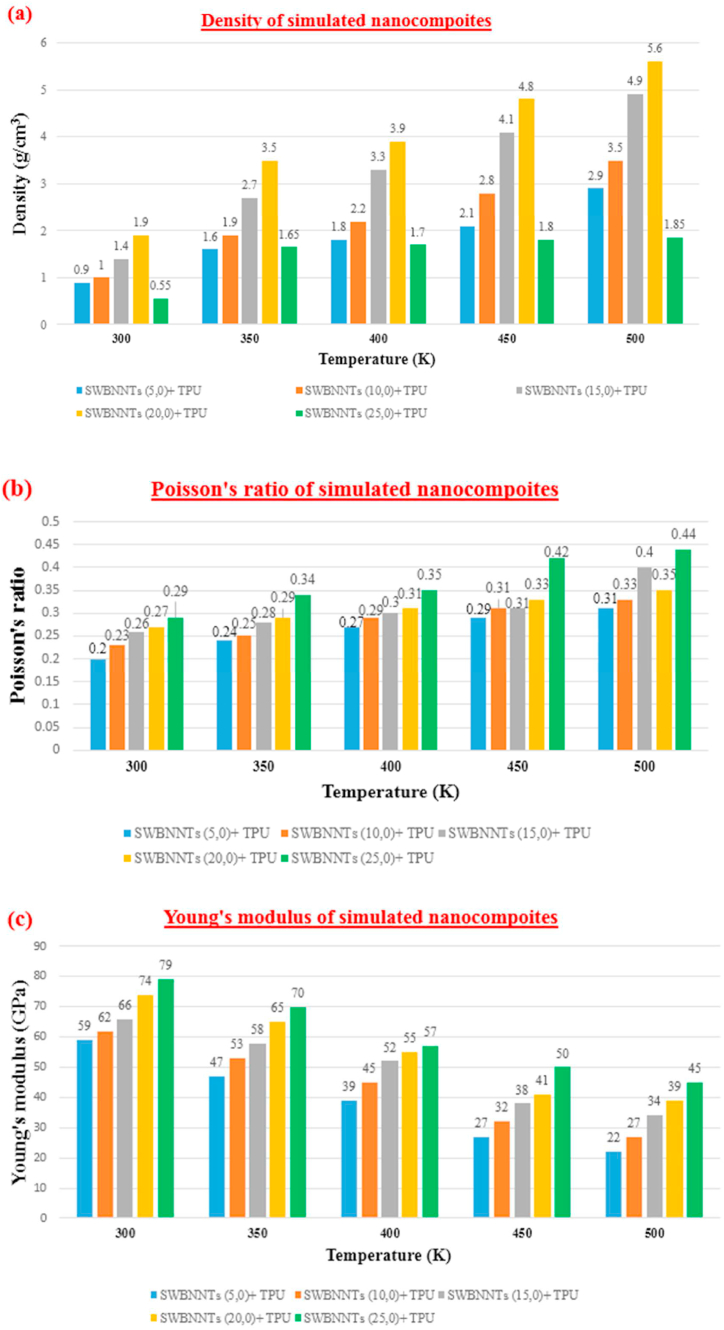

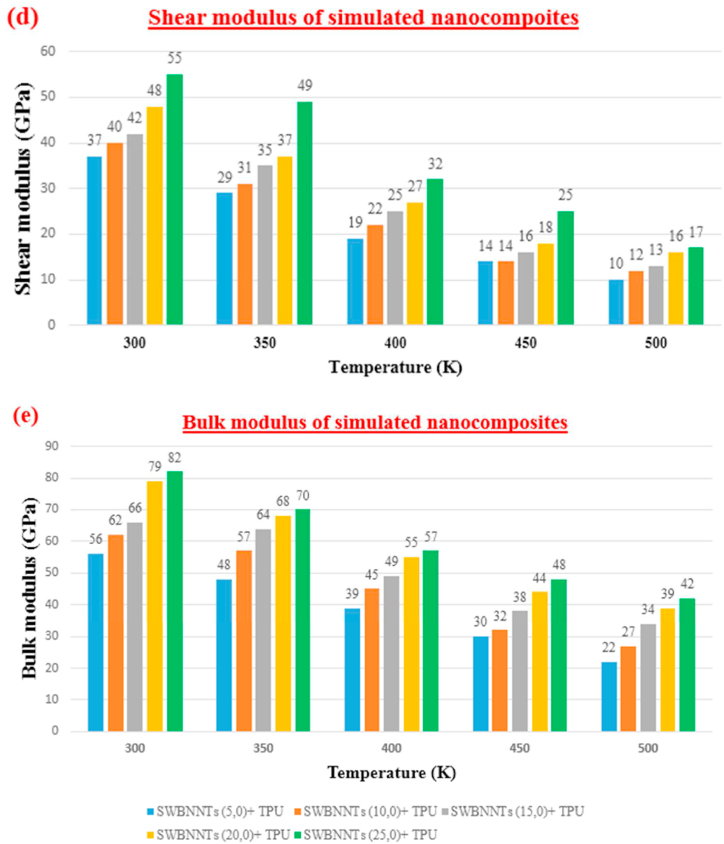
Chart depicting the Physical and Mechanical Characteristics of SWBNNTs-TPU **(a):**Density **(b):** Poisson's ratio **(c):** Young's modulus **(d):** Shear modulus **(e):** Bulk modulus.

Illustrated in Fig. 7, there is an initial rise in the physical property, specifically density, followed by a decrease at 500 K. Furthermore, as temperature increases and molecular motion becomes more pronounced, all mechanical properties, such as Poisson's ratio, Young's modulus, Shear modulus, and Bulk modulus, exhibit a declining trend. Density increases from SWBNNTs (5,0)-TPU to SWBNNTs (20,0)-TPU and then the density decreases due to approaching the melting point of the polymer. At temperature 300, 350,400, 450, and 500 K, physical and mechanical properties are obtained. Density for SWBNNTs (5,0)- TPU is 0.9, 1.6, 1.8, 2.1, and 2.9 g/cm^3^ respectively. And also density for SWBNNTs (25,0)- TPU is 0.55, 1.65, 1.70, 1.80, 1.85 g/cm^3^ respectively. Poisson's ratio for SWBNNTs (5,0)- TPU is 0.20, 0.24, 0.27, 0.29, 0.31 respectively. After that Poisson's ratio for SWBNNTs (25,0)- TPU is 0.29, 0.34, 0.35, 0.42, and 0.44 respectively. For example, for SWBNNTs (25,0) at the temperature 500 K, Poisson's ratio becomes approximately 1.51 times more than SWBNNTs (25,0) at the temperature 300 K. Young's modulus for SWBNNTs (5,0)- TPU is 59, 47, 39, 27, and 22 GPa respectively. And also Young's modulus for SWBNNTs (25,0)- TPU is 79, 70, 57, 50, and 45 GPa respectively. For SWBNNTs (25,0) at the temperature 500 K, Young's modulus becomes approximately 1.75 times less than SWBNNTs (25,0) at the temperature 300 K. Shear modulus for SWBNNTs (5,0)- TPU is 37, 29, 19, 14, and 10 GPa respectively. And also Shear modulus for SWBNNTs (25,0)- TPU is 55, 49, 32, 25, and 17 GPa respectively. For SWBNNTs (25,0) at the temperature 500 K, Shear modulus becomes approximately 3.23 times less than SWBNNTs (25,0) at the temperature 300 K. Finally Bulk modulus for various rang of temperatures are obtained. Bulk modulus for SWBNNTs (5,0)- TPU is 56, 48, 39, 30, and 22 GPa respectively. And finally Bulk modulus for SWBNNTs (25,0)- TPU is 82, 70, 57, 48, and 42 GPa respectively. For SWBNNTs (25,0) at the temperature 500 K, Bulk modulus becomes approximately 1.95 times less than SWBNNTs (25,0) at the temperature 300 K.

## 4 Conclusion

This research investigated and foretold the impact of elevated temperatures (ranging from 300 K to 500 K) on simulated nanocomposites reinforced with SWBNNTs and its consequent influence on both mechanical properties (including Young's modulus, Poisson's ratio, shear modulus, and bulk modulus) and physical properties (specifically, density). These simulations were conducted using the Molecular Dynamics (MDs) method and Universal Force Field.

The MDs method was applied to simulate nanocomposite models involving five distinct scenarios, featuring SWBNNTs of different chiralities, namely (5, 0), (10, 0), (15, 0), (20, 0), and (25, 0), serving as the reinforcing agents within a common matrix of thermoplastic polyurethane (TPU).

The most noteworthy findings are as follows:

1- Density increased from SWBNNTs (5,0)-TPU to SWBNNTs (20,0)-TPU and then the density decreases due to approaching the melting point of the polymer. The highest density of this nanocomposite was recorded for SWBNNTs (20,0)-TPU at 500 K which is equal to 5.6 g/cm^3^.

2 Mechanical properties (Young's, Shear, and Bulk moduli) decreased dramatically with increasing temperature.
3 For SWBNNTs (25,0)-TPU at the temperature of 500 K, Young's modulus becomes approximately 1.75 times less than SWBNNTs (25,0) at the temperature of 300 K.
4 For SWBNNTs (25,0) at the temperature of 500 K, Poisson's ratio becomes approximately 1.51 times more than SWBNNTs (25,0) at the temperature of 300 K.
5 For SWBNNTs (25,0) at the temperature of 500 K, Shear modulus becomes approximately 3.23 times less than SWBNNTs (25,0) at the temperature of 300 K.
6 For SWBNNTs (25,0) at the temperature of 500 K, Bulk modulus becomes approximately 1.95 times less than SWBNNTs (25,0) at the temperature of 300 K.
7 To check the accuracy of the results, diagram of RDF were obtained by Materials studio software.

## Data availability statement

Upon request, data will be accessible and provided.

## CRediT authorship contribution statement

**Somayeh Tavasolikejani:** Writing – original draft, Validation, Software. **Ashkan Farazin:** Writing – review & editing, Validation, Software, Methodology.

## Declaration of competing interest

The authors declare that they have no known competing financial interests or personal relationships that could have appeared to influence the work reported in this paper.
